# Speaking of Dementia: How to Refer to Dementia in Racial-Ethnic Minority Community-Facing Communications

**DOI:** 10.1093/geroni/igab046.1782

**Published:** 2021-12-17

**Authors:** Zachary Baker, Tetyana Shippee, Joseph Gaugler

## Abstract

What do you call “dementia”? In academic writing, researchers often chose the inclusive, “Alzheimer’s Disease and Alzheimer’s Disease Related Dementias (AD/ADRD)”. When referring to the people experiencing dementia, the person-centered language: “persons living with dementia (PLWD)” is preferred. This is a welcome departure from the antiquated disease-centered language of “dementia patients” or “the demented”. Still, AD/ADRD and PLWD may be less fitting in community-facing education or participant recruitment. For instance, community-facing materials may benefit from choosing terms like “memory loss”, “issues related to memory or aging”, or “changes in ability, behavior, or judgment”. In this symposium we present a range of viewpoints focused on how to refer to “dementia” in community-facing materials/conversations. These viewpoints include those of several racial and ethnic groups (i.e., African Americans, African Immigrants, American Indians, Asians, Hispanics/Latinos/as/x/e, and Whites). We also include viewpoints from people interfacing with many different diseases that cause dementia (i.e., Alzheimer’s disease, dementia with Lewy bodies, Early-onset Alzheimer’s disease, and Parkinson’s disease dementia) because of the different manifestations of dementia that can arise from those diseases. Viewpoints were gathered through 1) a nation-wide community advisory board, 2) community conversations with African Immigrants, 3) a national effort to increase the representation of Hispanics/Latinos/as/x/e PLWD in AD/ADRD research, and 4) eight community projects exploring the African American AD/ADRD experience. These talks will present possible terms to use within groups, considerations to increase inclusiveness, issues with translation into native languages, considerations surrounding symptoms that may be most recognizable to community members, and stigmatized terminology.

